# Case Report: A rare case of combined branch retinal vein occlusion and branch retinal artery occlusion

**DOI:** 10.3389/fmed.2026.1775574

**Published:** 2026-02-26

**Authors:** Dahu Wang, Yuyan Zhang, Hong Li

**Affiliations:** 1 Department of Ophthalmology, Longhua Hospital Affiliated to Shanghai University of Traditional Chinese Medicine, Shanghai, China; 2 Longhua Clinical Medical College, Shanghai University of Traditional Chinese Medicine, Shanghai, China

**Keywords:** branch retinal artery occlusion (BRAO), branch retinal vein occlusion (BRVO), anti-VEGF (vascular endothelial growth factor), laser photocoagulation, macular edema (ME)

## Abstract

**Significance:**

The combination of branch retinal vein occlusion (BRVO) and branch retinal artery occlusion (BRAO) is extremely rare. At present, the pathogenic mechanism underlying the occurrence of combined BRVO and BRAO remains unclear. Prompt treatment of macular edema (ME) secondary to combined BRVO and BRAO with anti-vascular endothelial growth factor (anti-VEGF) therapy and laser photocoagulation can achieve a satisfactory prognosis.

**Purpose:**

This report presents a rare case of ME secondary to combined BRVO and BRAO treated with anti-VEGF agents and laser photocoagulation.

**Case report:**

A 59-year-old man presented with a 3-month history of blurred vision in the left eye. The patient had a 5-year history of diabetes and hypertension. On examination, the best-corrected visual acuity (BCVA) was 20/20 in the right eye and 20/125 in the left eye, and the intraocular pressure in both eyes was within the normal range. Slit lamp examination revealed unremarkable anterior segments in both eyes, except for lens opacity. According to the fundus examination findings, a diagnosis of ME secondary to combined BRVO and BRAO in the left eye was established. The central macular thickness (CMT) in the left eye was 302 μm. The patient subsequently underwent angiography-guided sectoral laser photocoagulation and received intravitreal injections of anti-vascular endothelial growth factor (anti-VEGF) agents in the left eye. After 3 months of treatment, the BCVA in the left eye improved to 20/40, the CMT decreased to 182 μm, and the intraretinal fluid was resolved. Thereafter, the patient did not return to the clinic for further ophthalmic examinations.

**Conclusion:**

Combined BRVO and BRAO is an uncommon and extremely rare retinopathy. Intravitreal injection of anti-VEGF drugs and laser photocoagulation are effective treatments for ME secondary to combined BRVO and BRAO. In addition, systemic evaluation and close monitoring of cardiovascular risk factors should not be overlooked.

## Introduction

Ocular vascular occlusive disorders collectively represent one of the leading causes of visual disability among middle-aged and older populations, with significant visual morbidity and systemic associations (1). In recent years, retinal vascular occlusions affecting both the arterial and venous systems have been reported, usually presenting with sudden, painless diminution of vision (2–8). In particular, the combination of branch retinal artery occlusion (BRAO) and branch retinal vein occlusion (BRVO) is an exceedingly rare event.

Although BRVO can lead to widespread capillary non-perfusion (CNP), combined BRAO and BRVO should not be mistaken for ischemic BRVO, as well-demarcated, wedge-shaped areas of complete capillary dropout are not a feature of ischemic BRVO (8). In addition, macular edema (ME) is the leading cause of vision impairment in patients with RVO (1, 9, 10). At present, anti-vascular endothelial growth factor (anti-VEGF) agents have become the first-line therapy for ME secondary to RVO (10). Therefore, in this case, angiography-guided sectoral laser photocoagulation and intravitreal injections of anti-VEGF agents were used to treat retinal ischemia and ME secondary to combined BRAO and BRVO.

## Case report

### Initial examination

**Figure 1:**
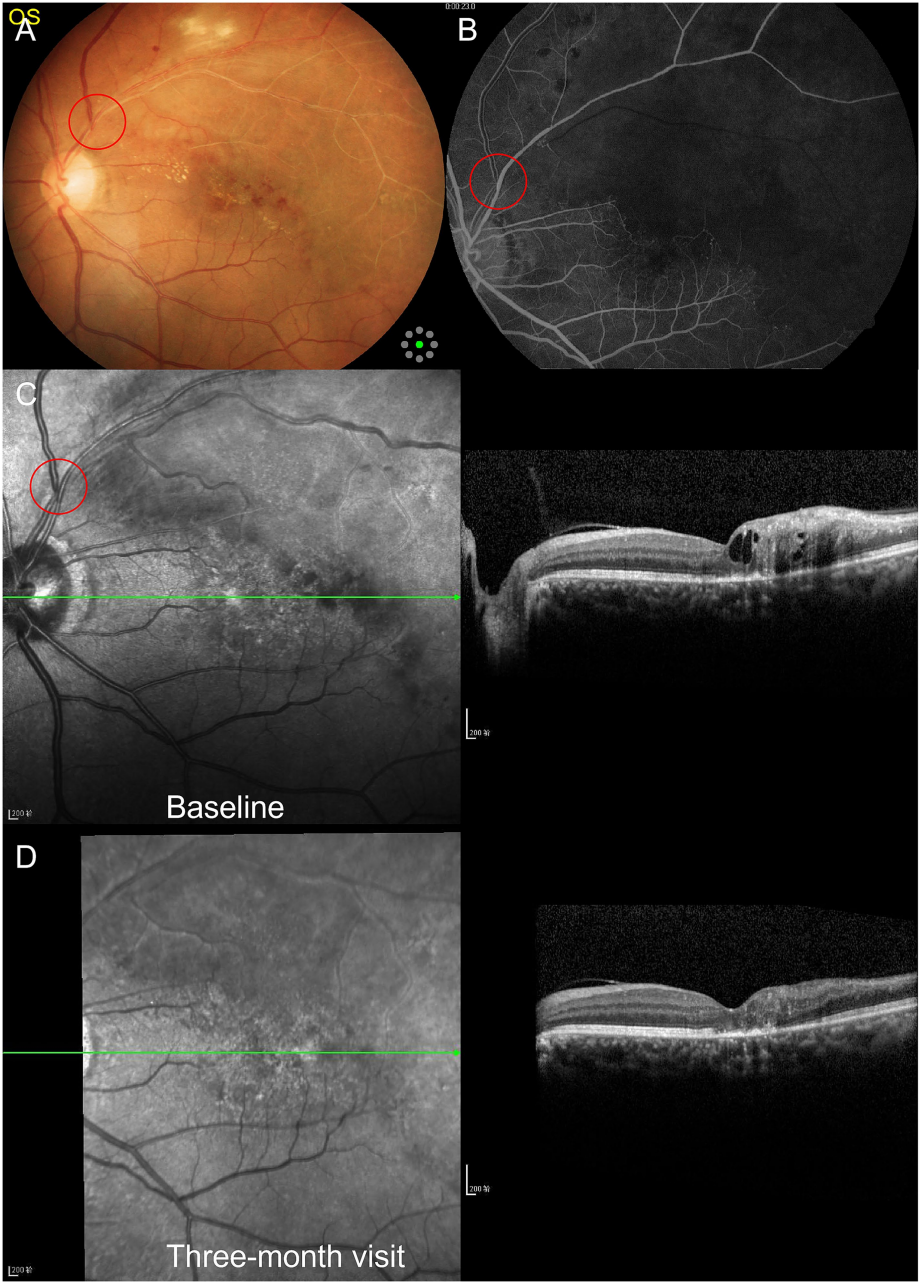
Multimodal imaging of the left eye before and after treatment. Red circles indicate the arteriovenous crossing sites.

A 59-year-old man presented with a 3-month history of blurred vision in the left eye. On examination, the best-corrected visual acuity (BCVA) was 20/20 in the right eye and 20/125 in the left eye, and the intraocular pressure in both eyes was within the normal range. Slit lamp examination revealed unremarkable anterior segments in both eyes, except for lens opacity. Color fundus photography revealed flame-shaped and blot retinal hemorrhages, cotton-wool spots, macular edema, and hard exudates. In addition, the retinal vein diameter and tortuosity, the arteriovenous crossing site, and the vascular sheath in the superotemporal quadrant were assessed (Figure 1). Fluorescence angiography revealed delayed filling of the involved artery and marked delay in venous filling, along with extensive areas of CNP (Figure 1). Optical coherence tomography (OCT) of the lesions in the left eye showed hyperreflectivity of the inner retinal layers and cystoid macular edema, with a central macular thickness (CMT) of 302 μm (Figure 1).

The patient had a 5-year history of diabetes and hypertension, and blood pressure (BP) and blood glucose levels were not monitored regularly. Subsequently, a panel of medical examinations was performed, including chest X-ray, electrocardiogram (ECG), magnetic resonance imaging (MRI) of the brain, carotid artery ultrasound, BP measurement, complete blood count (CBC), blood glucose, low-density lipoprotein cholesterol (LDLC), total cholesterol, triglycerides, homocysteine levels, and assessment of inflammatory and infectious disease markers. Except for poorly controlled arterial hypertension, with average blood pressure readings of approximately 140–160/100–110 mmHg, higher random blood glucose levels ranging from 8.5 to 13.0 mmol/L, dyslipidemia, carotid plaque, and common carotid artery intima-media thickening with less than 50% internal carotid artery stenosis, no other significant abnormalities were observed. Based on the above evaluations, a diagnosis of ME secondary to combined BRVO and BRAO in the left eye was made.

### Follow-up visits

Based on the management of systemic diseases, the left eye underwent angiography-guided sectoral laser photocoagulation and received monthly intravitreal injections of 2 mg/0.05 mL aflibercept. After 3 months of treatment, the BCVA in the left eye improved to 20/40, the CMT decreased to 182 μm, and the intraretinal fluid was resolved (Figure 1). Thereafter, the patient did not return to the clinic for further ophthalmic examinations.

## Discussion

Simultaneous retinal vascular occlusion affecting both the vein and artery is a rare occurrence and can present in various permutations, such as the combination of central retinal vein occlusion (CRVO) and central retinal artery occlusion (CRAO), CRVO and BRAO, BRVO and CRAO, BRVO and BRAO, and cilioretinal artery occlusion (CLRAO) and CRVO (2–8, 11–31). Among them, the combination of CRVO and CRAO is relatively more common (12–25). This report describes a rare case of combined BRVO and BRAO. Although ischemic BRVO can also cause extensive areas of CNP, fluorescein angiography and OCT examinations are valuable for establishing the correct diagnosis of combined BRVO and BRAO at an early stage.

Many systemic comorbidities, including hypertension, diabetes, hyperlipidemia, hyperhomocysteinemia, atherosclerosis, hyperviscosity syndromes, blood disorders, infections, tumors, pulmonary arterial hypertension, systemic vasculitis, and autoimmune diseases, can cause combined vascular occlusion (1–8, 11–13, 16–27, 29–31). The most commonly reported systemic associations are diabetes, hypertension, and dyslipidemia (1–8, 11, 12, 23, 31). In our case, these factors contributed to the occurrence of combined BRVO and BRAO.

The pathogenic mechanism underlying the occurrence of combined BRVO and BRAO remains unclear. Sengupta et al. (8) postulated that BRVO might be the initial event, resulting from compression of the vein by an atherosclerotic artery at an arteriovenous crossing site, leading to turbulent blood flow, dynamic obstruction, and even thrombus formation or mechanical blockage. If this situation is severe, a sudden increase in intravenous pressure exceeding the systolic BP may lead to the transmission of “back-pressure” to the arterial circulation, resulting in impaired arterial perfusion and the development of BRAO (8). In our case, the fundus images also appear to support this mechanism.

Lee et al. (32) reported that the incidence of neovascularization elsewhere (NVE) and/or neovascularization of the disc (NVD) was 21.4% in patients with BRVO combined with major arterial insufficiency. Sengupta et al. reported a case series of NVE, NVD, and neovascular glaucoma (NVG) secondary to combined BRVO and BRAO (8, 30). In addition, foveal neovascularization associated with combined BRVO and BRAO has also been reported (7). Management strategies for combined BRVO and BRAO are aimed at reducing posterior segment ischemia, improving vision, and decreasing the neovascular drive. Among the cases reported in the literature, Christodoulou et al. (31) described a patient with NVD, NVE, and ME secondary to combined BRAO and BRVO in the right eye, who was treated with angiography-guided sectoral laser photocoagulation and intravitreal therapy, achieving a good visual outcome. In general, the prognosis of combined BRAO and BRVO depends on the ischemic index and macular perfusion status, and earlier identification and appropriate treatment may result in better visual outcomes (8, 31).

In this case, we administered intravitreal injections of anti-VEGF agents and performed retinal laser photocoagulation to treat retinal ischemia and ME and to prevent the development of NVE, NVD, rubeosis iridis, and NVG. Three months later, visual acuity and ME in the left eye improved. Unfortunately, after that, the patient refused to return to the clinic for further examinations. Long-term monitoring would have added greater value to this case. Therefore, further studies are needed to investigate the management of recurrent ME secondary to combined BRAO and BRVO. In addition, systemic evaluation and close monitoring of cardiovascular risk factors are essential (2, 11, 12, 23, 31).

## Conclusion

Combined BRVO and BRAO is an uncommon and extremely rare retinopathy. Multimodal imaging, such as fluorescein angiography and OCT, is valuable for establishing the correct diagnosis, as well as for monitoring disease progression and treatment response. Combined BRVO and BRAO can be effectively managed with retinal laser photocoagulation and anti-VEGF agents. In addition, systemic evaluation and close monitoring of cardiovascular risk factors should not be overlooked.

## Data Availability

The original contributions presented in the study are included in the article/supplementary material, further inquiries can be directed to the corresponding authors.
